# Cellular accumulation of lipofuscin in the heart: implications in health and disease

**DOI:** 10.1007/s00418-026-02501-w

**Published:** 2026-06-25

**Authors:** Amy Li, Sean Lal, Gerald J. Shami, Kenneth S. Campbell, Eddie Wisse, Filip Braet

**Affiliations:** 1 Health Hub, Torrens University Australia, Sydney, NSW Australia; 2 Department of Rural Clinical Sciences, La Trobe Rural Health School, La Trobe University, Melbourne, Victoria Australia; 3 School of Medical Sciences, The University of Sydney, Sydney, NSW Australia; 4 Australian Centre for Microscopy and Microanalysis, The University of Sydney, Sydney, NSW Australia; 5 School of Medical Sciences (Molecular Biomedicine), The University of Sydney, Sydney, NSW Australia; 6 Division of Cardiovascular Medicine, University of Kentucky, Lexington, KY 40536 USA; 7 Division of Nanoscopy, Multimodal Molecular Imaging Institute, University of Maastricht, Maastricht, The Netherlands

**Keywords:** Aging pigment, Cellular stress, Chronic disease, Histopathological marker, Primary and secondary lysosomes, Lipofuscin structure–function, Volume electron microscopy

## Abstract

Lipofuscin is a subcellular pigmented granule that has long been considered a sign of wear-and-tear and aging. This granule cannot be degraded but may be diluted by cell division. In the heart, lipofuscin can be easily identified in cardiomyocytes where proliferative capacity is limited, and to a lesser extent in other cardiac cell types, by their characteristic morphological features. They appear as yellow–brown granules in histopathology sections reflecting their lipid origins, and as heterogenous membrane-bound bodies containing a mix of lipid and electron-lucent content in transmission electron microscopy. Lipofuscin was originally considered a mere byproduct of age-related cell senescence but studies over the past three decades have indicated that its role and mechanism of formation may be far more complex. In this review, we examine how lipofuscin is formed and its implications in aging and diseases of the heart. While the focus of this review is on lipofuscin in the two most predominate cell types of the heart, cardiomyocytes and fibroblasts, we also touch on the presence of lipofuscin in the vasculature in certain disease phenotypes that are directly relevant to cardiovascular health.

**Supplementary Information:**

The online version contains supplementary material available at 10.1007/s00418-026-02501-w.

## Introduction

Lipofuscin is a non-degradable aging pigment that progressively accumulates in post-mitotic cells. It is a byproduct of inefficient cellular recycling and accumulates in lysosomes. In the heart, cardiomyocytes are particularly vulnerable to lipofuscin accumulation due to their high metabolic activity and limited regenerative potential resulting in extended exposure to oxidative stress and reactive oxygen species (ROS). Lipofuscin increases with age and is therefore viewed as a biological maker of the aging myocardium. Despite being traditionally considered as an inert granule, there is mounting evidence to suggest that lipofuscin may have adverse effects impacting normal cell function, particularly by interfering in lysosomal-mediated autophagy.

Lysosomes, being highly acidic vesicles, play a central role in recycling cellular waste. Primary lysosomes, formed from the trans-Golgi network, contain a suite of hydrolytic enzymes. These enzymes are inactive until their fusion with autophagic vesicles containing defective macromolecules or organelles. This process transforms primary lysosomes into secondary lysosomes where macromolecules are broken down into monomers that can be recycled by the cell. However, macromolecules can be oxidatively modified or cross-linked which makes them resistant to lysosomal degradation. These materials accumulate as residual bodies within secondary lysosomes, contributing to the formation of lipofuscin granules. Lipofuscin preferentially accumulates in long-lived post-mitotic cells such as cardiomyocytes as it cannot be diluted by cell division and autophagic recycling efficiency declines with aging.

This thematic contribution explores the role of lipofuscin in cardiac tissue in the context of aging and disease. We discuss the conditions under which lipofuscin can be induced, in vitro and in vivo, providing important insights into its formation. We also examine its involvement in cardiovascular diseases and its implication for heart health. Moreover, at the end of this contribution, all of these literature findings are summarized in a schematic depicting the subcellular and molecular pathways identified to date. Throughout the literature, lipofuscin is typically described interchangeably using the terms ‘pigment’, ‘granule’, and ‘vesicles’ which causes confusion. These terms reflect the historical characterization where ‘pigment’ refers to the optical properties of these structures, which accumulate to form ‘granular’ structures observed by microscopy, and the ‘vesicles’ denotes the lipofuscin within membrane-bound lysosomes. For clarity, lipofuscin will be referred to as granules throughout this article, as this term acutely reflects its observable form in cardiac cells.

## Lipofuscin formation: history and development

**Fig. 1:**
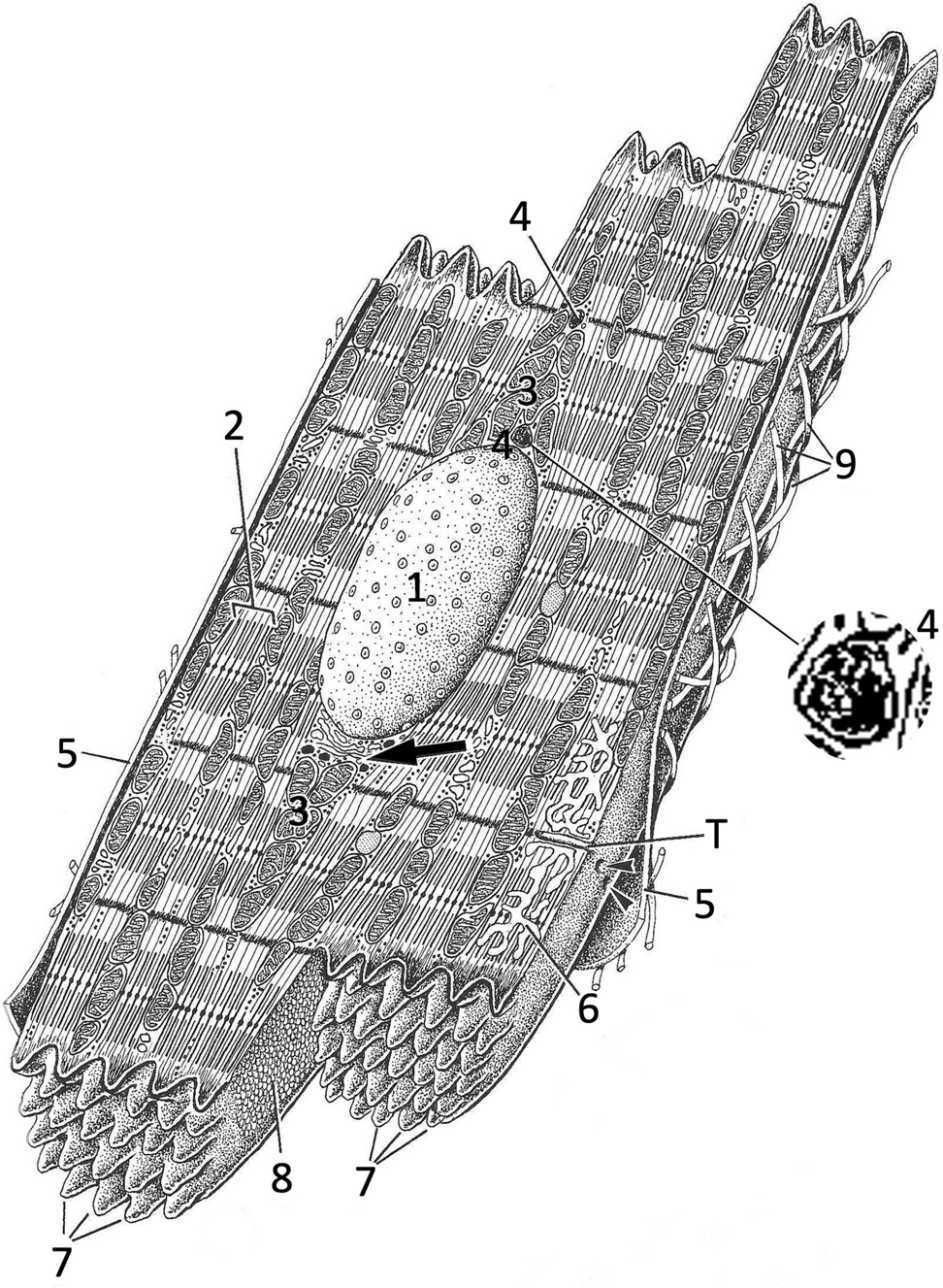
Three-dimensional drawing of the interior of an isolated cardiac muscle cell. *Legend*: 1, nucleus; 2, myofibrils; 3, mitochondria; 4, lipofuscin pigments laying close to mitochondria; 5, basal lamina; 6, smooth endoplasmic reticulum; 7, intercalated discs which interlock with adjacent cardiomyocytes; 9, collagen and reticular microfibrils; T, T-tubule. The arrow denotes perinuclear laying osmiophilic membrane-bound pigments that contain cardiodilatin. Note, main illustration is drawn to scale, × 6000. (Reprinted and modified with permission from Springer-Verlag (Krstić 1978): Copyright 1978–1979)

**Fig. 2:**
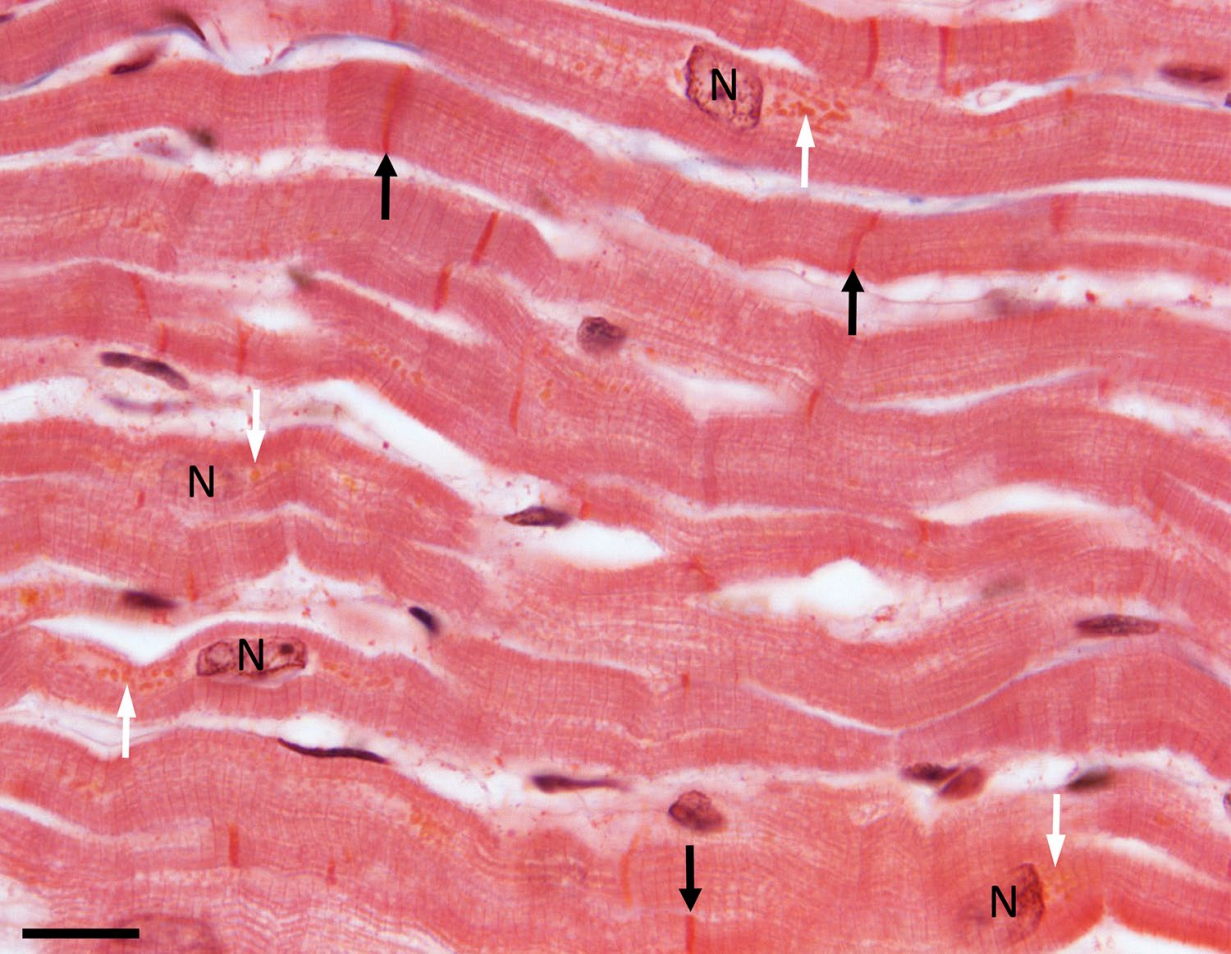
Wide-field light optical image of a paraffin-embedded section of human heart tissue stained with Mallory’s trichrome. White arrows point to brown–orange pigments representing lipofuscin inclusions within the cytoplasm of cardiomyocytes. These pigments typically locate around the nucleus (N) of the heart muscle cells. Black arrows, intercalated discs. Scale bar, 20 μm

**Fig. 3:**
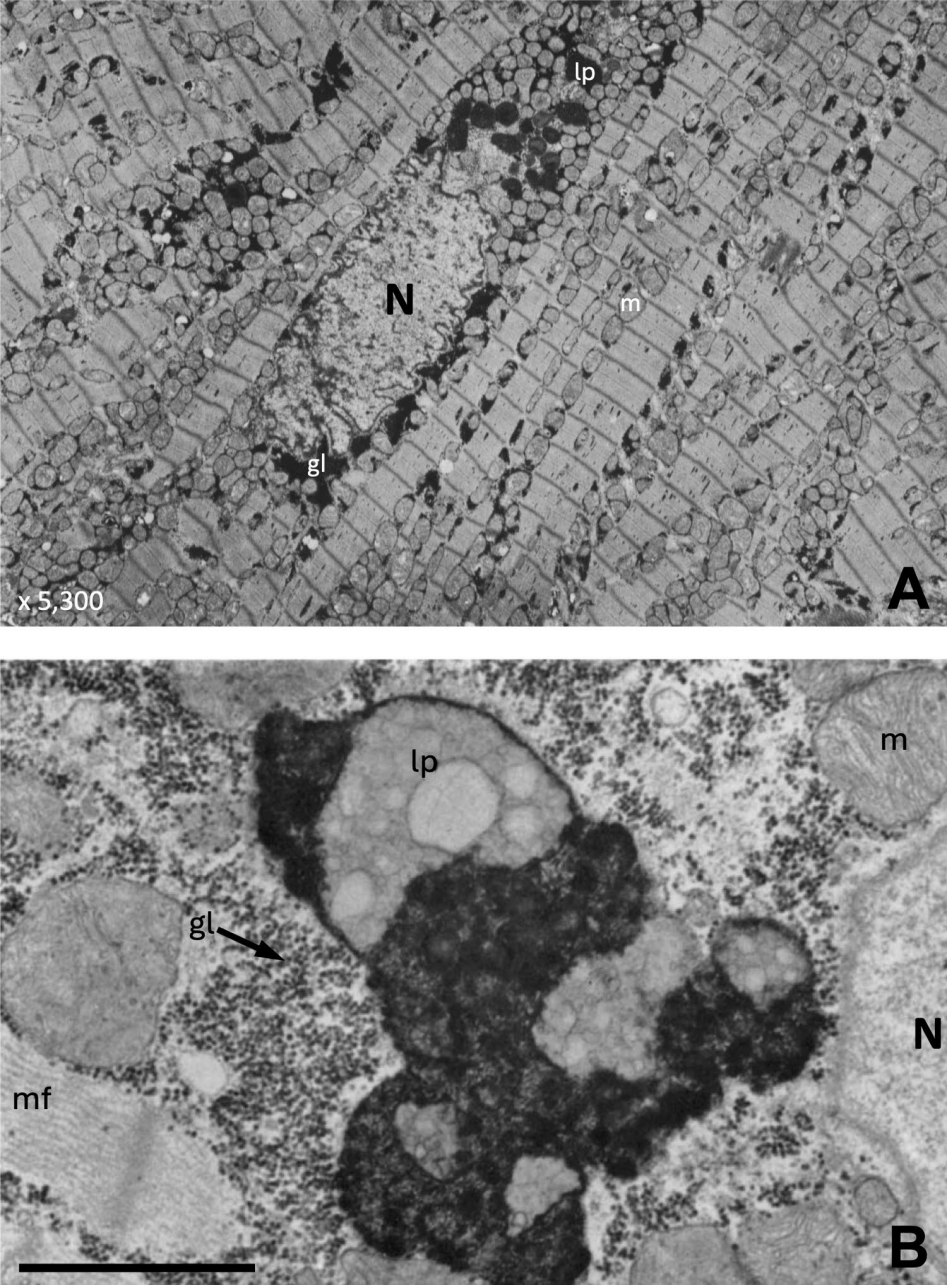
Transmission electron microscopy images of human (**A**) and dog (**B**) cardiac muscle tissue, both showing lipofuscin pigment pigments (lp). **A** Low-magnification overview image of a hypertrophied cardiac muscle cell showing numerous intact myofibrils that are separated by mitochondria (m). The centrally located nucleus (N) has convoluted membranes and is surrounded by a myofibril-free area that is filled with mitochondria, glycogen (gl) particles, and lipofuscin pigment pigments (lp). Magnification, × 5300. (Reproduced and lettering modified with permission [ID-600188592] of Elsevier Science & Technology Journals in the format Journal/Magazine via Copyright Clearance Center—Fig. 1: American Journal of Pathology by Elsevier Science & Technology Journals; Maron et al. 1975). **B** High-power magnification image of a lipofuscin pigment (lp) in the sarcoplasmic core of an atrial muscle fiber. *Legend*: N, nucleus; gl, glycogen particles (black arrow); mf, myofibrils. Scale bar, 1 μm. (Reproduced and lettering modified with permission of Rockefeller University Press [ID-600188740] in the format Journal/Magazine via Copyright Clearance Center—Fig. 13: Journal of Cell Biology by Rockefeller University Press; Jamieson and Palade 1964)

The accumulation of lipofuscin in heart tissue has long been recognized as a key morphological marker of cellular and organ stress, a significance repeatedly underscored in medical and histology textbooks (e.g., Fig. 1). Lipofuscin in heart tissue appears as a yellow–brown pigment in a bright-field microscope (Fig. 2). In transmission electron microscopy (TEM), lipofuscin appears as electron-dense, granular aggregates within lysosomes 0.5–5 µm in diameter (Fig. 3). They are mainly confined to the perinuclear area of the cardiac myocytes (Skepper and Navaratnam 1987). There is an extensive body of clinical, fundamental, and experimental literature on heart lipofuscin, including studies in cultured cardiac cells, which we discuss in the following sections.

One of the earliest descriptions of lipofuscin in cardiac muscle cells was given by Heidenreich and Siebert (1955). The authors isolated lipofuscin without chemical treatment that might denature the native properties of the pigment. This analysis revealed 20% lipid, 12% protein, and a high level of magnesium, aluminum, and iron. Lipofuscin also showed weak protease and esterase activity which the authors attributed to properties absorbed rather than native components of the pigment. This was an early indicator of what we now know, that lipofuscin is a pigment found within secondary lysosomes containing proteases (cathepsin) and esterase (De Duve et al. 1955).

Others speculated that lipofuscin is formed from the degradation of intracellular organelles such as Golgi apparatus, mitochondria, or the endoplasmic reticulum as summarized in Terman et al. (2010). Biochemical and TEM studies by Bjoerkerud and Cummins (1963) found low levels of cytochrome oxidase and ATPase activity in lipofuscin which ruled out their localization within mitochondria or endoplasmic reticulum. Further, purified lipofuscin showed oxygen uptake independent of mitochondrial respiration. Early observations of oxygen reserves and the presence of carotenoids, myoglobin, and other respiratory enzymes led to the hypothesis that lipofuscins may act as an energy store that can be called upon when stressed (Karnaukhov et al. 1973).

Subsequent tissue culture experiments identified the precise conditions under which lipofuscin forms within cells. Oxidative stress and free radical generation in mitochondria are directly related to ambient oxygen concentration which may also drive lipofuscin accumulation. To examine this relationship, rat cardiac myocyte cell cultures were exposed to 5% (hypoxia), 20% (normoxia), and 40% (hyperoxia) oxygen (Sohal et al. 1989b). Autofluorescent lipofuscin granules appeared after 7 days. After 12 days, the abundance of autofluorescent pigments per cell was 50% and 250% higher in normoxic and hyperoxic conditions respectively, compared to hypoxia. Lipofuscin in hyperoxic cells appear to be larger and more structurally complex in keeping with the greater proportion of pigments. Further, spectral analysis of lipofuscin pigments revealed emission peaks between 510 and 530 nm (Sohal et al. 1989b), similar to reported wavelengths in glial cells (Collins and Brunk 1976).

Next, Terman and Brunk examined the contributions of oxidative stress and lysosomal proteolysis to the formation of lipofuscin in rat neonatal cardiac cells (Terman and Brunk 1998b) and human fibroblasts (Terman and Brunk 1998a; Terman et al. 1999). Fibroblasts constitute a major cell type of the myocardium and play a critical role in maintaining the extracellular matrix, which underscores their relevance in studying lipofuscin in cardiac tissue. In human fibroblasts, the combined effects of hyperoxia-induced oxidative stress and leupeptin (a protease inhibitor which reduces lysosomal proteolysis) over a 2-week period increased the concentration of lipofuscin by more than three times the sum of the effects of each condition in isolation (Terman and Brunk 1998a). These insights highlight a key mechanism for lipofuscin formation in vitro. Under sustained oxidative stress, incompletely degraded autophagic material undergoes oxidative modifications over time to form nondegradable, autofluorescent granules.

Terman and Brunk also discovered that lipofuscin granules formed under oxidative stress could rapidly disappear if the stress was relieved within 1 week or less of exposure. In contrast, after extended exposure, lipofuscin persisted in cells even when those cells were reintroduced to normal culture conditions. This indicates that when lipofuscin undergoes oxidation and becomes autofluorescent, it also becomes resistant to degradation (Terman and Brunk 1998b, a). The fibroblasts laden with lipofuscin are also more sensitive to oxidative stress (Terman et al. 1999). Acute exposure to naphthazarin, which induces severe oxidative stress, causes over 60% of the fibroblasts to undergo apoptosis, preferentially those with high lipofuscin content. This effect is linked to the membranes of lipofuscin-laden lysosomes, which are prone to rupture when exposed to naphthazarin. This study also confirmed the subcellular localization of lipofuscin through the presence of acridine orange and cathepsin D, demonstrating that it resides in active lysosomes rather than in non-lysosomal organelles, as previously thought.

The impact of oxidative stress on lipofuscin formation has been further examined using glutathione and Polbax. Glutathione, an oxygen scavenger, and Polbax, an antioxidant, both provided protection against oxidative stress in cardiac cells which reduced lipofuscin concentrations (Gao et al. 1994; Terman and Brunk 2002). Although neither compound completely prevented lipofuscin accumulation, their presence slowed its formation, highlighting the contribution of oxidative processes to granule development.

Together, these studies highlight three properties pertinent to the formation and impact of lipofuscin: (1) the transition from degradable lipofuscin in autophagosomes to undegradable lipofuscin in older lysosomes requires oxidative stress over a prolonged period; (2) lipofuscin depletion was found in confluent, proliferative fibroblasts but not in cardiomyocytes with limited proliferative capacity; and (3) high lipofuscin content sensitizes cells to apoptosis under stress. While mitochondrial reactive oxygen species are the primary source of stress, transition metals such as iron can independently induce oxidative damage and cytotoxicity (Höhn et al. 2010). Iron both contributes to lipofuscin formation and incorporates into existing granules, generating free radicals that further impair cell viability. Collectively, these findings indicate that lipofuscin is likely more than a passive bystander.

The specific component of lipofuscin responsible for generating or responding to oxidative stress remains unclear. Liquid chromatography–mass spectrometry (LC–MS) analysis from isolated human cardiac lipofuscin and myocardial lysates showed similar amino acid content except proline was enriched from 8% to 14% in lipofuscin (Baldensperger et al. 2024). The authors suggest that proline promotes protein cross-links that contribute to lipofuscin’s resistance to degradation. It is also possible that proline is sequestered into lipofuscin preventing its normal function in the cytosol which then impairs protein aggregation (Fisher 2006). These observations support the idea that lipofuscins may be formed in the cytosol and subsequently imported into lysosomes (Höhn and Grune 2013; Höhn et al. 2012).

Several metals found in the myocardium, including redox-active elements, are present at higher concentrations in lipofuscin than in the surrounding tissue. Calcium and iron were the most abundant at eight-fold (5000 mg/kg) and three-fold (500 mg/kg) higher in lipofuscin, respectively (Baldensperger et al. 2024). This raises the question of whether lipofuscin can catalyze oxidation by virtue of its composition. Earlier work provides clues on the effects of iron on heart cells, which become sequestered into secondary lysosomes also containing lipofuscin (Marzabadi et al. 1988). As previously discussed, optimal conditions for lipofuscin formation in cardiomyocytes involves prolonged exposure to hyperoxia. The addition of 30 µM of ferric iron further increased the lipofuscin levels in as little as 6 days, while desferrioxamine, an iron-chelating agent, could reduce lipofuscin levels but required a concentration of 50 µM to show significant effects. Note, concentrations above 50 µM desferrioxamine were cytotoxic. These findings indicate that iron and oxidative stress seen under hyperoxia cumulatively amplify the lipofuscin inside cells.

Ethanol, a known contributor of oxidative stress and cardiac damage, was also examined for its effects on lipofuscin accumulation. In isolated rat cardiomyocyte cultures, ethanol was evaluated across a range of concentrations from 0 to 12.5 mM (Sohal et al. 1989a). Low-dose 3.1 mM ethanol consistently reduced the lipofuscin present in cells by an average of 10–25% across oxygen conditions, with similar reductions in lipofuscin across all ethanol concentrations under normoxia. In hyperoxia, however, ethanol exhibited dose-dependent effects where low-dose ethanol reduced while high-dose 12.5 mM ethanol enhances lipofuscin content. Contextually, prolonged hyperoxia was nearly as effective in increasing lipofuscin content in control cells as ethanol.

These findings led Sohal et al. to propose that low-dose ethanol (3.1 mM) may attenuate lipofuscin accumulation. To assess the functional consequences, cardiomyocyte contractility (contractions/min or beats per minute, bpm) over a 21-day period was measured (Sohal et al. 1989a). Under normoxic conditions, ethanol had minimal effects on contractility with rates remaining stable at approximately 60 bpm until day 15, followed by a gradual decline through to day 21. Under hyperoxic conditions, ethanol treatment extended cell survival by approximately 16% compared to control. While cells in both ethanol and control conditions reached peak contractility of 120 bpm at day 6 under hyperoxia, contractile function declined more rapidly thereafter than under normoxic conditions.

Overall, these findings provide insights into the impact of oxygen saturation on contractile function. In comparison, the reduction in lipofuscin observed at 3.1 mM ethanol was modest relative to the accumulation induced by prolonged hyperoxia. It is therefore unlikely that such a small reduction in lipofuscin alone can confer meaningful cardioprotective effects against heart rhythm disturbances. Nevertheless, this study was one of the first reports linking lipofuscin accumulation to functional changes in contractility.

To conclude this section, and as a perfect transition to the next section on aging, we highlight the seminal ultrastructural study by George Palade’s group. Jamieson and Palade were among the first to use TEM to quantify and describe in minute detail the various electron-dense pigmented granules and other subcellular inclusions present in the different anatomical regions of heart muscle tissue across biological ages (Jamieson and Palade 1964). The authors described irregularly shaped granular structures, which they identified as lipofuscin granules or residual bodies of varying electron density, measuring approximately 0.5 µm in diameter and present in both atrial and ventricular muscle cells. These were often located in the perinuclear region and interspersed among the Golgi complex and mitochondria. In addition to lipofuscin, the authors also focused on spherical granules measuring approximately 0.3–0.4 µm in diameter, exhibiting uniform electron density, situated mainly at the poles of the nucleus, and present in atrial but not ventricular myocardium. At that time, the role of these smaller, uniformly shaped granules was unclear. However, their distinct morphology suggested that they were unlikely to be lipofuscin. This was further supported by their high concentration in neonatal cells, followed by a reduction in size and abundance with age, as well as their lack of acid phosphatase activity and their positive reaction with reserpine, which is consistent with an endocrine granule identity. It was only later that it became evident that these uniform granules contained atrial natriuretic peptide, a hormone that plays a key role in blood pressure regulation. Conversely, Jamieson and Palade noted a significant increase in the number of lipofuscin granules with age. For completeness, we recommend comparing the atrial electron micrographs from cats (McNutt and Fawcett 1969) with the ventricular cardiomyocyte electron micrographs from hamsters (Skepper and Navaratnam 1987), as these two TEM studies clearly address the different types of granular inclusions and their micro-anatomical distribution.

**Fig. 4:**
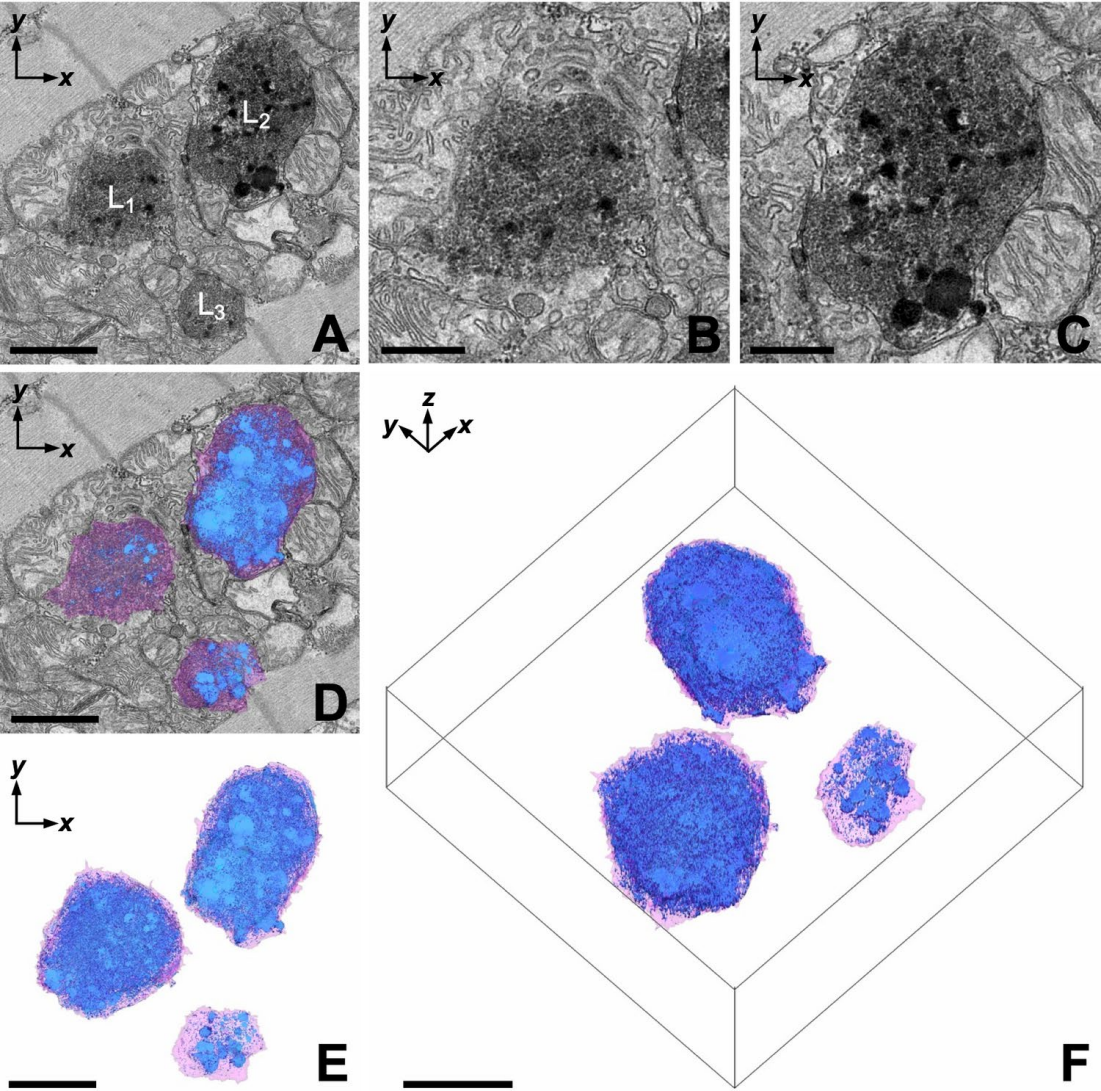
Serial-section transmission electron microscopy reconstruction of lipofuscin in human heart tissue. **A** Intermediate-magnification electron micrograph showing three membrane-bound lipofuscin granules (L_1_, L_2_, L_3_) within the field of view. Scale bar, 1 µm. **B**, **C** High-magnification image data corresponding to the selected L_1_ and L_2_ regions in image** A**, respectively. The micrographs reveal amorphous electron-dense granular material of varying size. Scale bars, 500 nm. **D** Rendered 3-D model overlaid on the 2-D micrograph shown in** A**, displaying the limiting membrane (magenta) and granular material (blue). Scale bar, 1 µm. **E**, **F** 3-D model of image **D** from different angles illustrating the fine structural organization of the lipofuscin granules, showing contents with varying density and size. Scale bars, 1 µm. See also, Supplementary Information—Video

One can envision that, had the authors mentioned in the previous paragraph had access at that time to modern three-dimensional (3-D) electron microscopy (Eisenstein 2023), they would have been able to map these granular inclusions throughout the entire volume of cardiomyocytes. Indeed, determining the precise volumetric context and connectivity of lipofuscin with other cellular structures is crucial for understanding how these granules interact with, and potentially affect, cell state and fate. This statement is underpinned by the elegant study by Vue et al. (2023), who applied volume electron microscopy to reveal nanometer-scale 3-D mitochondrial changes in aging cardiomyocytes. Figure 4 is an example from our group illustrating the added value that volume electron microscopy can bring in obtaining detailed 3-D structure–function insights of lipofuscin granules. This iso-surface-rendered model provides a holistic view by following the course of the delineating membranes and their interactions with nearby organelles and other subcellular complexes, which cannot be resolved using classical electron microscopy (see also, Supplementary Information — Video). Collecting insights such as these under different conditions may guide the development of targeted therapies to slow or reverse cardiac aging.

## Lipofuscin in the aging heart

Aging is a major risk factor for cardiovascular disease, and age-related alterations in the cardiac ultrastructure likely contribute to the development and progression of these pathologies. Among these changes, lipofuscin accumulates progressively with age reflecting the gradual decline in autophagic processes (Nakano and Gotoh 1992). The initial appearance of lipofuscin during adolescence parallels the metabolic shifts associated with sexual maturation and the biological transition into adulthood (Gerstbrein et al. 2005; Nakano et al. 1990). This was also verified in humans where lipofuscin was virtually absent in hearts younger than 10 years of age (Goyal 1981; Strehler et al. 1959).

In humans, lipofuscin was found to accumulate at a rate of 0.61–0.67% per myocardial volume per decade (Goyal 1981; Strehler et al. 1959). Accumulation did not differ significantly with respect to anatomical region, sex, race, or the interval between autopsy time and fixation (Strehler et al. 1959), although inconsistent sampling across anatomical locations limited definitive conclusion. In a comparative study of aging hearts, Nakano et al. (1993) quantified lipofuscin in crab-eating monkeys and compared the findings with mammals of varying sizes including tree shew, dogs, primates, and humans (Nakano et al. 1993). Larger animals, which generally exhibit lower cellular metabolic rates and longer lifespans, demonstrated correspondingly slower lipofuscin accumulation. When normalized to species-specific lifespan, lipofuscin accumulates at a relatively uniform and linear annual rate of less than 1% per myocardial volume per 10% of lifespan (Nakano et al. 1993).

Regional variation in cardiac lipofuscin distribution was clarified by Li et al. (2021). Examination of all four anatomical chambers in short-lived rat and long-lived oxen revealed consistent findings irrespective of lifespan. Lipofuscin content increased with age and was associated with cellular senescence as demonstrated by marked β-galactosidase staining in aged, lipofuscin-laden cells. Ventricular tissue contained more lipofuscin than atrial tissue. Although lipofuscin levels were minimal in conducting tissue under normal conditions (Li et al. 2021), deposits have been observed in transitional cells of the sinoatrial nodes and atrial cardiomyocytes of young patients (20–36 years) with inappropriate sinus tachycardia (Lowe et al. 1988). Overall, the findings of Li et al. indicate that lipofuscin accumulation reflects both aging and regional workload of the heart, with distribution patterns conserved across species.

Beyond chamber differences, lipofuscin accumulation also varies across heart layers, reflecting their distinct functions. The epicardial and endocardial layers of the ventricular myocardium were compared in rats aged between 2 and 24 months (Del Roso et al. 1991). While all layers showed increasing lipofuscin accumulation from 6 months of age, the highest levels were found in the endocardial layer. The aged endocardium had larger and brighter autofluorescent lipofuscin granules than those in the epicardium, suggesting aggregation. These findings reflect chamber-level differences, with the endocardium exhibiting higher metabolic activity and workloads (Pitoulis et al. 2020) analogous to the atria vs. ventricle.

To determine whether lipofuscin accumulation could be mitigated, aged 18-month-old rats were treated with rapamycin for 6 months (Li et al. 2021). Rapamycin has dual effects: it promotes autophagic processes and diminishes cellular senescence. Treatment increased autophagic structures and lysosomes, and reduced the abundance of defective mitochondria and residual bodies. Notably, autophagic and lysosomal structures were inversely correlated to intracellular lipofuscin levels. These findings suggest rapamycin can reduce lipofuscin content by approximately 30%, though it does not eliminate these granules. This reduction may result from the degradation of newly formed lipofuscin as previously noted by Terman and Brunk (1998b, a). On the basis of current evidence, we postulate that rapamycin likely prevents the de novo accumulation of lipofuscin, but is unlikely to degrade pre-existing lipofuscin. While rapamycin is approved as an immunosuppressant, is used off-label in aging research, and has analogues that are used to target certain cancers, this drug can cause serious side effects, particularly in immunocompromised individuals (Li et al. 2014).

A recent study has further clarified the harmful effects of lipofuscin accumulation in the aged human myocardium (Baldensperger et al. 2024). This in vitro study found 95% of the native lipofuscin in fibroblasts were localized in lysosomes. Lysosomal localization dropped to 88% when fibroblasts were treated with isolated aged human or equine lipofuscin. Notably, the cytotoxic effects of isolated human lipofuscin were 15 times stronger than from equine hearts. Although mitochondrial content remained unchanged, lipofuscin increased mitochondrial ROS levels comparable to paraquat exposure. Lipofuscin loading also reduced lysosome numbers and compromised their membranes, neutralizing the optimal acidic environment required by the digestive lysosomal enzymes. Despite lysosomal membrane permeabilization, acid lipase, cathepsins B and D remained within the lysosomes with only cathepsin D activity showing a 70% reduction. Reduced cathepsin D levels were previously linked to the accumulation of autofluorescent lipofuscin pigments in retinal cells (Escrevente et al. 2021). This study highlights the interplay between lipofuscin, mitochondrial ROS, and lysosomal damage.

Given the interplay between lipofuscin, lysosomes, and ROS, mitochondrial dysfunction may play a key role in lipofuscin pathobiology in the aging myocardium. Lipofuscin accumulates with age and has been associated with impaired mitochondrial intermembrane potential indicative of mitochondrial damage (Terman et al. 2004). Impaired autophagy is a hallmark of the aging heart and this process, as we have described above, independently affects both lipofuscin deposition and mitochondrial structure and function. While lipofuscin may not directly cause mitochondrial dysfunction, the resulting oxidative stress likely creates a feedback loop that promotes further lipofuscin formation. Thus, the presence of lipofuscin is a reasonable downstream litmus of cells that have been functioning inefficiently over an extended period.

## Implications of lipofuscin in cardiovascular disease

The link between lipofuscin and heart disease has produced conflicting observations. Several studies investigating this relationship reported limited associations that appear unrelated to aging (Kakimoto et al. 2019; Yokota 1980; Strehler et al. 1959), while others have identified lipofuscin in animal models of heart disease (Tatariunas 1991) and in the human myocardium (Soleiman et al. 2008; Zakliczynski et al. 2009). Lipofuscin in cardiovascular disease will be discussed below.

Elevated lipofuscin levels have been observed in hypertrophic cardiomyopathy, although no differences were identified between degenerated vs. non-degenerated hypertrophied cells (Maron et al. 1975). Lipofuscin deposition has also been reported in left ventricular biopsies from patients with type 2 diabetes (Regan et al. 1977), dilated cardiomyopathy (Gerdes et al. 1995; Nozynski et al. 2013), and ischemic cardiomyopathy (Nozynski et al. 2013), where it was generally described as intracellular deposits. In the aforementioned conditions, lipofuscin colocalized with a subset of advanced glycation end-products (AGE) (Nozynski et al. 2013) which were subsequently shown to impair lysosomal acidification and proteolysis (Bou-Teen et al. 2026). Together, these studies suggest AGE, which accumulates in cardiomyocytes over time, may contribute to lipofuscin formation.

Myocardial samples from patients diagnosed with various cardiomyopathies were examined for the apoptosis marker, caspase cleaved cytokeratin-18 (ccCK-18), and lipofuscin (Soleiman et al. 2008). Patients with congestive, hypertrophic and ischemic cardiomyopathy were all positive for ccCK-18, which colocalized with lipofuscin at levels higher than age-matched controls. The greatest elevation in ccCK-18 was detected in lipofuscin-loaded lysosomes within cardiomyocytes in ischemic cardiomyopathy patients. The concurrent increase in ccCK-18 and lipofuscin in cardiomyopathies may suggest diminished lysosomal capacity, but to better understand the implications, the source of elevated ccCK-18 levels requires further evaluation.

Until now, lipofuscin accumulation in cardiomyocytes was regarded as an indicator of autophagic decline and impaired intracellular waste processing. However, a study of 200 endocardial biopsies of individuals under 40 years of age found high lipofuscin abundance was strongly associated with marked improvement in cardiac function 12 months later (Parson et al. 2012). The mechanism underlying this association remains unclear, but it is possible that enhanced autophagy in young hearts accumulates lipofuscin as a byproduct of increased cellular repair.

In a separate study on endomyocardial tissue of post-transplant hearts, elevated lipofuscin levels were predictive of cardiac allograft vasculopathy at 12 months—a common mortality outcome (Zakliczynski et al. 2009). Additional indicators of cellular rejection included enlarged endothelial cells with vacuolization and lymphocyte infiltration of the arteriolar wall. Together, these studies suggest that the effect of lipofuscin is context-dependent, with vascular pathology being more prominent than myocardial involvement. One possible explanation is the presence of inflammatory cells that can release and amplify oxidative stress signals (Steven et al. 2019).

The aortic valves in mice fed an atherosclerotic diet also saw significant deposits of lipofuscin (Mehrabian et al. 1991). Across anatomical regions of the aorta, lipofuscin was limited to the aortic leaflets facing the ventricles. This side of the aortic leaflets experiences high-shear flow and was lined with macrophages but lacked lipids or lipoproteins. In contrast, lipofuscin was absent in regions exposed to low shear force. High shear stress may damage cells, leading to macrophage recruitment independent of lipoprotein deposits, whereas atherosclerotic plaques require both macrophages recruitment and excessive lipoproteins (Hou et al. 2023). Lipofuscin, which forms under cellular stress, may further promote macrophage recruitment to the atherosclerotic microenvironment.

Beyond the aorta, lipofuscin was also found in the atherosclerotic lesions of the coronary and carotid arteries. In patients with carotid stenosis, lipofuscin was present within macrophages surrounding the necrotic cores of advanced atherosclerotic plaques (Cromheeke et al. 1999). Lipofuscin was absent in the arteries during early-stage atherosclerosis, including intimal thickening and fatty streak formation, as verified by autofluorescence and Ziehl–Neelsen staining. Instead, it colocalizes with regions of dense macrophage infiltration expressing NOS (nitric oxide synthase) II, a major driver of oxidative stress. These findings reinforce the earlier study which identified lipofuscin in plaques but not in areas of intimal thickening (Mitchinson et al. 1985). Lipofuscin was also observed in endothelial and smooth muscle cells of atherosclerotic plaques of the aorta (Perrotta 2018). TEM and X-ray analysis demonstrated the colocalization lipofuscin with iron in macrophages and smooth muscle cells of aortic plaques (Lee et al. 1998). Collectively, these studies suggest that lipid peroxidation employing various sources, NOS II or iron, facilitates lipofuscin formation.

Studies on diseased vasculature make the distinction between lipofuscin and ceroids, with ceroids being the preferred nomenclature in vascular pathology (Cromheeke et al. 1999; Mitchinson et al. 1985; Perrotta 2018). In these publications, ceroid was defined as “osmophilic deposits with a fingerprint pattern” (Perrotta 2018) and as a “multi-laminated membranous structure” (Cromheeke et al. 1999). Lipofuscin on the other hand was described as electron-dense granules limited by a lipid membrane, often containing lipid vacuoles scattered throughout the structure (Cromheeke et al. 1999; Mitchinson et al. 1985; Perrotta 2018).

Human coronary arteries obtained from cardiomyopathy heart transplants had extensive lipid deposition in endothelial cells, independent of cholesterol levels (Joris et al. 1998). Lipofuscin was associated with lipids of varying levels of oxidation. Two forms of lipofuscin are present which may reflect their origins: membrane-free lipofuscin derived from oxidation of lipoproteins within lipid droplets, and membrane-bound lipofuscin derived from secondary lysosomes. The former was abundant in endothelial cells and often oxidized from the periphery of the droplet, possibly due to free radicals within the endothelial cytoplasm. A recent paper on rat hearts also reported lipofuscin in capillary endothelial cells, cardiomyocytes, and other interstitial cells (Yang et al. 2023).

Thus far, we have discussed the relationship between lipofuscin in cardiac disease. Here, we describe one final case report on the histopathology of the heart caused by ephedra toxicity in heart failure (Chen-Scarabelli et al. 2005). Ephedra, with the stimulant ephedrine as its active ingredient, is a traditional herbal supplement used for weight loss, respiratory relief, and performance enhancement. Pathology revealed widespread accumulation of lipofuscin predominately around the perinuclear region of cardiomyocytes alongside myofibrillar loss consistent with activation of apoptosis markers, caspase-3 and caspase-9. In the heart, its mechanism of action remains to be elucidated but ephedrine in the liver triggers oxidative stress-induced mitochondrial dysfunction resulting in hepatotoxicity (Lee et al. 2017).

## Conclusion

**Fig. 5:**
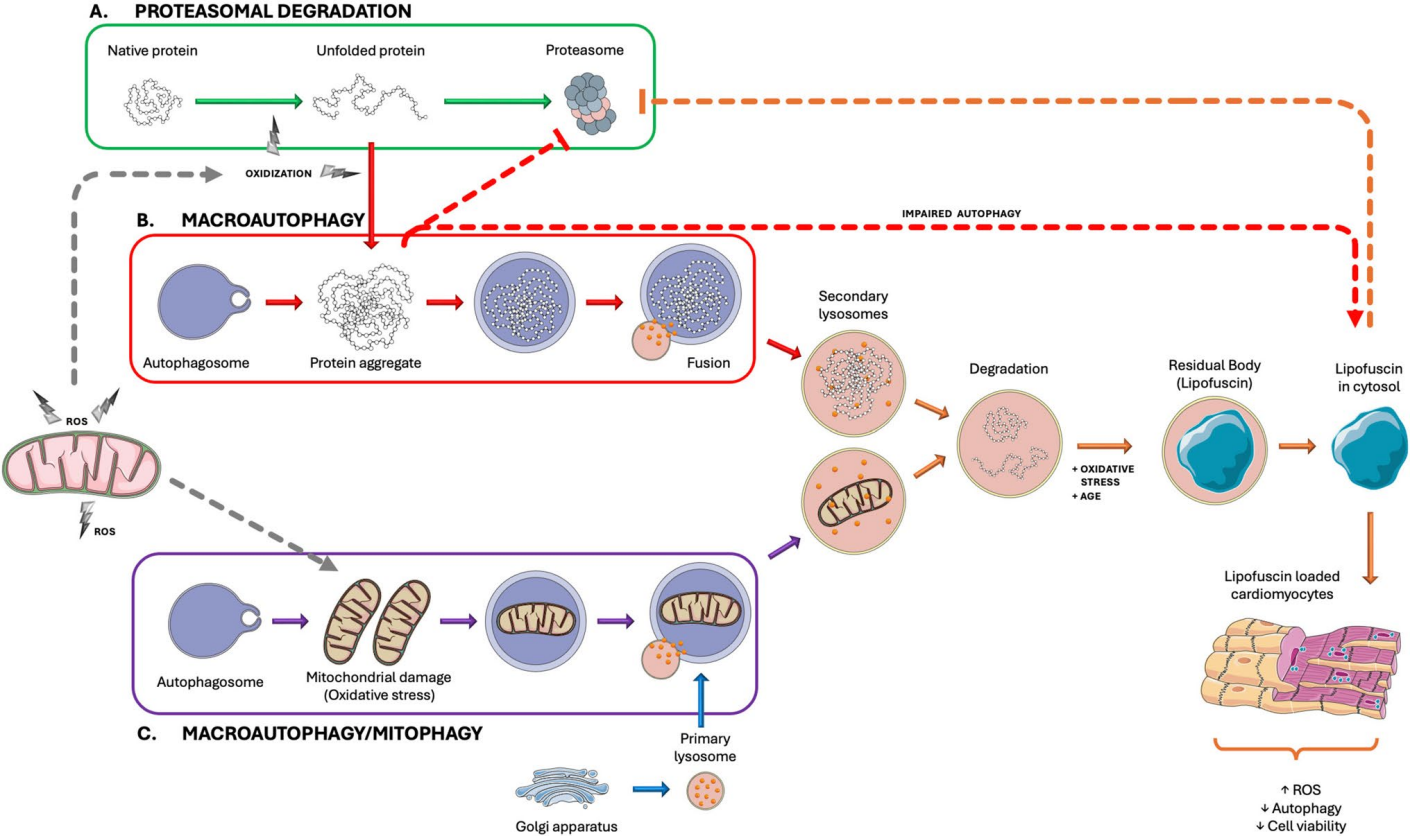
Schematic depicting the three main pathways of lipofuscin formation. **A** Recycling of defective macromolecules (e.g., proteins, lipids, carbohydrates, nucleic acids, etc.). In this case, proteins are typically recycled by proteasomal degradation pathway (green line box). With age and oxidative stress, however, this process becomes less efficient resulting in the cross-linking and aggregation of the proteins to be recycled. Those aggregates can impair the proteosome function resulting in enhanced lipofuscin formation (red dotted lines). **B** Protein aggregates (red line box) and **C** organelles damaged by oxidative stress (purple line box) are recycled by macroautophagy. In the latter, autophagosomes engulf the large, damaged organelles structures which then fuse with primary lysosomes derived from the Golgi apparatus. Once fused, the lysosomal enzymes are activated, forming secondary lysosomes which actively degrade their internal contents (orange, arrows). However, with added oxidative stress and age, the degradation becomes inefficient resulting in the accumulation of residual bodies (lipofuscin) within cells which ultimately impacts cell viability and health. Lipofuscin may impair normal proteasomal processes (orange, dotted line). Alternatively, enlarged mitochondria which bypass mitophagy due to their size may further contribute to oxidative stress (gray, dotted arrow). ROS, reactive oxygen species

In this review, we highlight and discuss key findings in the formation of lipofuscin and its role in the aging and diseased cardiovascular system (Table 1). Lipofuscin has long been regarded as a harmless marker of the aging cell, although more recent studies have suggested that its accumulation reflects cell senescence, enhanced oxidative stress, impaired lysosomal recycling with significant implication for cell death. The heart displays a complex pattern of lipofuscin deposition reflecting regional variations. Lipofuscin accumulation seems to be modulated by factors such as blood flow and metabolic activity, which vary significantly across different regions and layers of the heart. Importantly, lipofuscin has conflicting and distinctive roles in cardiovascular disease. Significant accumulation was noted in the diseased heart vasculature associated with immune infiltration, and may be an early indication of allograft rejection, but its presence was also associated with long-term improvement in cardiac function in young hearts. The precise mechanisms and conditions through which lipofuscin forms in the human heart, and ultimately how it influences heart health and function, remain elusive. Further research is needed to delineate the pathways through which lipofuscin impacts cellular function and explore its potential as a therapeutic target, either through direct modulation of lipofuscin or by targeting the associated intracellular pathways and organelles involved. We have summarized the putative mechanisms of lipofuscin formation in the heart in Fig. 5, as revealed by this literature study.

**Table 1:** Brief overview of key literature on lipofuscin in cardiac cells

Species/model	Key finding	Imaging/technique	References
**Aging**			
Human	One of the earliest methodological descriptions of lipofuscin isolation from heart muscle. Chemical analysis and enzymatic activity were measured	LM & FM	Heidenreich and Siebert 1955
Human & equine	Isolation of human lipofuscin from aged heart tissue. Quantified the metal (ICP-MS) and amino acid (LC–MS) content of lipofuscin relative to heart tissue levels	LM, flow cytometry, LC–MS & ICP-MS	Baldensperger et al. 2024
Hamster	The relationship between cellular organelles and the formation of lipofuscin using acid phosphatase staining. Atrial and ventricular cardiomyocytes were examined	TEM	Skepper and Navaratnam 1987
Rat	Investigates impact of ferric iron on lipofuscin production	FM & TEM	Marzabadi et al. 1988
Rat	Characterized the conditions of oxidative stress and ethanol exposure under which lipofuscin accumulates in cardiac cells cultured from Sprague Dawley rats. The ethanol study was among the first to associate lipofuscin accumulation with altered contractility	LM, FM & TEM	Sohal et al. 1989a, 1989b
Rat	Investigated aging and transmural variation of the left ventricle	FM	Del Roso et al. 1991
Primate	Aging, metabolic rate and lipofuscin in formalin-fixed hearts of crab-eating monkeys. Lipofuscin levels were also compared to mammals of various sizes	FM	Nakano et al. 1993; Nakano and Gotoh 1992
Rat	In neonatal cardiac cells, depleting the naturally occurring antioxidant glutathione with buthionine sulfoximine increased lipofuscin accumulation after 14 days	FM & TEM	Gao et al. 1994
Rat	Neonatal cardiac cells. Examined the role of reducing intralysosomal protein degradation via application of leupeptin	LM, FM & TEM	Terman and Brunk 1998b
Rat	Neonatal cardiac cells. Polbax is an antioxidant derived from grain pollen containing superoxide dismutase. It was effective in reducing lipofuscin formation in a dose-dependent manner	FM & TEM	Terman and Brunk 2002
Rat	Neonatal rat ventricular myocytes. Describes the relationship between oxidative stress, mitochondrial damage, and lipofuscin generation in aged cells	FM & TEM	Terman et al. 2004
Rat & ox	Studied lipofuscin cardiac senescence and autophagy. Regional variations were also touched on in mammal hearts with different lifespan. mTOR may serve as a therapeutic agent against lipofuscin accumulation	LM & FM	Li et al. 2021
Mice	Aged cardiomyocytes accumulate advanced glycation end products in mitochondria which impairs lysosomal function and contributes to lipofuscin	FM & TEM	Bou-Teen et al. 2026
**Disease**			
Human	Lipofuscin was identified in sinoatrial node of young patients with inappropriate sinus tachycardia	TEM	Lowe et al. 1988
Human	Compares lipofuscin in arteries and veins derived from patients with heart disease	TEM	Joris et al. 1998
Human	Lipofuscin in the vasculature of atherosclerotic plaques	LM, FM, TEM, & XR	Cromheeke et al. 1999; Mitchinson et al. 1985; Perrotta 2018; Lee et al. 1998
Human	In cardiomyopathy explants, lipofuscin was associated with caspase-cleaved cytokeratin-18 in the lysosome	LM & FM	Soleiman et al. 2008
Human	Lipofuscin predicts cardiac improvement	LM	Parson et al. 2012
Human	Lipofuscin is present in the failing heart irrespective of the type of heart failure. Lipofuscin co-occurs with diffuse but not granular advanced glycation end products	LM & FM	Nozynski et al. 2013
Mice	Lipofuscin is present in the valves of mice on high fat diets	LM	Mehrabian et al. 1991

The above table succinctly summarizes, in chronological order by species and year, hallmark ultrastructural and/or biochemical observations of lipofuscin (also known as aging pigment or ceroid) in cardiomyocytes

*FM* fluorescence microscopy, *FS* fluorescence spectroscopy, *LM* light microscopy, *NAFLD* non-alcoholic fatty liver disease, *TEM* transmission electron microscopy, *TEM* transmission electron microscopy, *XR* X-ray elemental analysis

## Supplementary Information

Below is the link to the electronic supplementary material.

Supplementary file1 (DOCX 859 KB)

Supplementary file2 (AVI 298411 KB)

## Data Availability

The data generated as part of Figs. 2 and 4, as well as the Supplementary Information, and/or analyzed in the current paper are available from the corresponding authors upon reasonable request.
