# Supplementary material for: Cellular accumulation of lipofuscin in the heart: implications in health and disease
Source: Histochem Cell Biol. 2026 Jun 25;164(1):51. doi: 10.1007/s00418-026-02501-w (PMC13303463; doi:10.1007/s00418-026-02501-w)
Supplement: Supplementary file 1 — Supplementary file1 (DOCX 859 KB) [file 418_2026_2501_MOESM1_ESM.docx]

**Supplementary Information**

**“Cellular Accumulation of Lipofuscin in the Heart –**

**Implications in Health and Disease”**

**in Histochemistry and Cell Biology**

Amy Li^1,2,3^*, Sean Lal^3^, Gerald J. Shami^4,5^, Kenneth S. Campbell^6^,

Eddie Wisse^7^, Filip Braet^4,5^*

*^1^Health Hub, Torrens University Australia, NSW Australia; ^2^Department of Rural Clinical Sciences, La Trobe Rural Health School, La Trobe University, Victoria, Australia; ^3^School of Medical Sciences, The University of Sydney, NSW Australia; ^4^Australian Centre for Microscopy & Microanalysis, The University of Sydney, NSW Australia;  ^5^School of Medical Sciences (Molecular Biomedicine), The University of Sydney, NSW Australia; ^6^Division of Cardiovascular Medicine, University of Kentucky, Lexington, KY, 40536, USA; ^7^Division of Nanoscopy, Multimodal Molecular Imaging Institute, University of Maastricht, Maastricht, The Netherlands*

***Corresponding authors**: Amy Li [amy.li@torrens.edu.au](mailto:amy.li@torrens.edu.au) | <https://orcid.org/0000-0001-5413-3771> & Filip Braet [filip.braet@sydney.edu.au](mailto:filip.braet@sydney.edu.au) | <https://orcid.org/0000-0002-5222-0895>


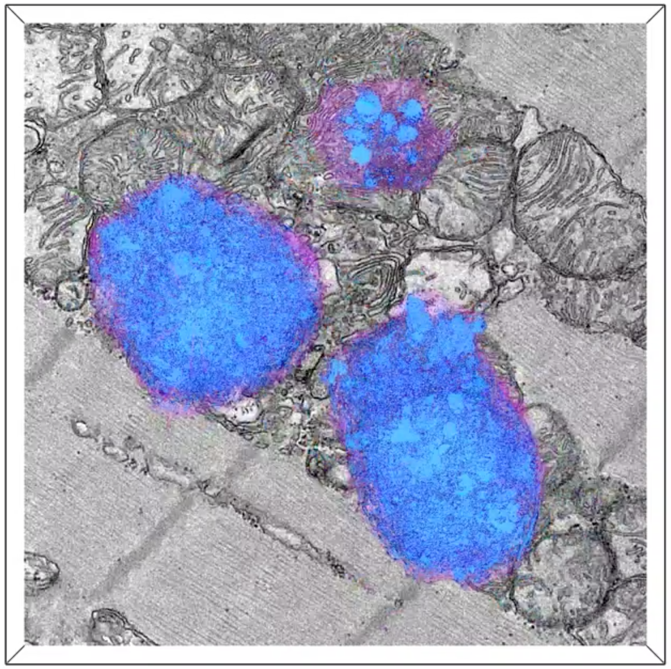


***Supplementary Video***. Animated volume-segmentation data extending the transmission electron microscopy image data presented in Figure 4. Note the fine, granular electron-dense material of varying density and size (blue) within the lipofuscin granules. The limiting membrane of the lipofuscin granules is shown in magenta within the model.
